# Modulation of osmotic stress-induced TRPV1 expression rescues human iPSC-derived retinal ganglion cells through PKA

**DOI:** 10.1186/s13287-019-1363-1

**Published:** 2019-09-23

**Authors:** Chih-Chien Hsu, Ke-Hung Chien, Aliaksandr A. Yarmishyn, Waradee Buddhakosai, Wen-Ju Wu, Tai-Chi Lin, Shih-Hwa Chiou, Jiann-Torng Chen, Chi-Hsien Peng, De-Kuang Hwang, Shih-Jen Chen, Yuh-Lih Chang

**Affiliations:** 1 Department of Ophthalmology, Taipei Veterans General Hospital, Taipei, 112 Taiwan; 2 Institute of Pharmacology, National Yang-Ming University, Taipei, 112 Taiwan; 3 School of Medicine, National Yang-Ming University, Taipei, 112 Taiwan; 4 Department of Ophthalmology, Tri-Service General Hospital and National Defense Medical Center, Taipei, 114 Taiwan; 5 Department of Medical Research, Taipei Veterans General Hospital, Taipei, 112 Taiwan; 6 Department of Ophthalmology, Shin Kong Wu Ho-Su Memorial Hospital and Fu-Jen Catholic University, Taipei, Taiwan; 7 Department of Pharmacy, Taipei Veterans General Hospital; Institute of Pharmacology, School of Medicine, National Yang-Ming University, Taipei, 112 Taiwan

**Keywords:** TRPV1, PKA, Osmotic stress, Human-induced pluripotent stem cells, hiPSC, Retinal ganglion cells, RGC

## Abstract

**Background:**

Transient receptor potential vanilloid 1 (TRPV1), recognized as a hyperosmolarity sensor, is a crucial ion channel involved in the pathogenesis of neural and glial signaling. Recently, TRPV1 was determined to play a role in retinal physiology and visual transmission. In this study, we sought to clarify the role of TRPV1 and the downstream pathway in the osmotic stress-related retina ganglion cell (RGC) damage.

**Methods:**

First, we modified the RGC differentiation protocol to obtain a homogeneous RGC population from human induced pluripotent stem cells (hiPSCs). Subsequently, we induced high osmotic pressure in the hiPSC-derived RGCs by administering NaCl solution and observed the behavior of the TRPV1 channel and its downstream cascade.

**Results:**

We obtained a purified RGC population from the heterogeneous retina cell population using our modified method. Our findings revealed that TRPV1 was activated after 24 h of NaCl treatment. Upregulation of TRPV1 was noted with autophagy and apoptosis induction. Downstream protein expression analysis indicated increased phosphorylation of CREB and downregulated brain-derived neurotrophic factor (BDNF). However, hyperosmolarity-mediated defective morphological change and apoptosis of RGCs, CREB phosphorylation, and BDNF downregulation were abrogated after concomitant treatment with the PKA inhibitor H89.

**Conclusion:**

Collectively, our study results indicated that the TRPV1–PKA pathway contributed to cellular response under high levels of osmolarity stress; furthermore, the PKA inhibitor had a protective effect on RGCs exposed to this stress. Therefore, our findings may assist in the treatment of eye diseases involving RGC damage.

## Background

Cell homeostasis and ion balance are pivotal for cell survival. The ion channels at the cell membrane function to equilibrate the electrolyte and ion gradients. Electrolytes are crucial for initiation and propagation of the membrane’s action potential, which is the shifting of electrical charge. This process is crucial for fluid and waste exchange and responding to nerve impulses. Furthermore, ionic imbalance may activate several vital enzymes, including protein kinases, phosphatases, and proteases, which leads to biochemical and physical changes as well as neuronal death. The electrolyte–ion imbalance affects extracellular fluid osmolarity. An acute change of osmolarity has been noted to affect the excitable properties of neurons in the brain.

Retinal ganglion cells (RGCs), a type of neurons located near the inner surface of the retina, can receive visual information from photoreceptors through bipolar cells and amacrine cells. They collect and transmit visual information from the retina to the brain in the form of action potential. RGC survival is critical for vision; however, these neurons are extremely sensitive to injury. Injury or disease, such as ischemic optic neuropathy, optic neuritis, glaucoma, or retinal ischemic diseases, may affect RGC axons in the optic nerve or RGC bodies and can lead to RGC death and cause significant vision loss. Hyperosmolarity, resulting from either hyperglycemia or dietary salt, is thought to be one of the pathological factors of intraocular inflammation. Furthermore, hyperglycemia-induced hyperosmolarity, occurring in diabetic retinopathy, was found to increase expression of ICAM-1 and VCAM-1 as well as reduce NO production [1]. These factors are known to be involved in angiogenesis and inflammation in diabetic microvasculopathy [2]. High intake of dietary salt is a major risk factor for systemic hypertension, which is associated with the development of age-related macular degeneration (AMD). Salt- or NaCl-induced high extracellular osmolarity in retinal pigment epithelium (RPE) cells was proven to trigger expression of VEGF-A, VEGF-D, and PIGF, as well as the secretion of PIGF-2, leading to angiogenesis in wet-type AMD. High extracellular NaCl has also been shown to stimulate NFAT5 expression in RPE [3, 4]. In addition, high-salt-induced hyperosmolarity can induce IL-6 and MCP-1 production and is associated with activation of the p38 MAPK, Akt, NF-κB, and NFAT-SGK1 pathways in intraocular inflammatory responses [5]. However, studies exploring the cellular response of RGCs to high-salt-induced osmolar stress are scant.

The members of the transient receptor potential vanilloid (TRPV) family are known to mediate the osmotic stress response of cells. The TRPV proteins are tetramer channels with each subunit comprising six transmembrane domains. Of the seven identified members (TRPV1-TRPV7) of this family, only two, TRPV1 and TRPV4, are recognized as osmotic sensors. TRPV1 mediates responses to a variety of stimuli, including osmotic stress, heat, pain, stretching, and chemical substances. TRPV1 functions as a nonselective cation channel and is widely expressed in various organs, such as brain, dorsal root ganglia, heart, liver, lungs, kidney, salivary glands, and sensory neurons [6, 7]. Initially, TRPV1 was characterized as a Ca^2+^ channel activated by capsaicin, the active component of hot chili peppers [8]. Most studied functions of TRPV1 are related to the pain pathway because of its involvement in sensory transmission from nociceptive neurons of the peripheral nervous system [9].

TRPV1 is also known to be involved in vision function. It is expressed in photoreceptors, bipolar cells, amacrine cells, glial cells, and RGCs of the retina, where it is involved in a complex modulatory mechanism among these cell types. Ca^2+^-influx-dependent RGC excitability is required for vision transduction from photoreceptors to the brain’s visual cortex; this is believed to be the core function of TRPV1 in the retina. TRPV1 activity was shown to be elevated after exposure to high hydrostatic pressure and found to be excitotoxic for cultured RGCs [10]. However, TRPV1 depletion also harms RGCs [11]. Thus, TRPV1 regulation in RGCs is critical for maintaining cell homeostasis and cellular response under stress conditions.

TRPV1 interacts with several cellular proteins and modulates several signal transduction pathways to regulate synaptic transmission. Recently, it was implicated in cell survival pathways involving autophagy and apoptosis. In retina development, TRPV1 plays a role in neuronal and synaptic maturation by modulating the MAPK signaling pathway [12]. In adult retinas, TRPV1-expressing neurons of the inner retinal layer were found to be involved in NMDA signaling and upregulation of nitric oxide synthases in retinal cell death [13]. NMDA-induced neuronal injury was shown to be prevented by the activation of TRPV1 channels [14]. TRPV1 was found to be potentially involved in neuroprotection. As shown in an animal model of ischemia–reperfusion brain injury, the activation and phosphorylation of Akt and mTOR associated with TRPV1 activation had a neuroprotective effect through increased expression of Golgi phosphoprotein 3 [15]. Naloxone, an opioid receptor antagonist, was found to reduce capsaicin-induced neuroprotection in brain injury, and morphine was found to lessen ischemia–reperfusion injury in the retina through TRPV1 activation [16]. However, the role of TRPV1 in high osmolarity-induced injury in RGCs is unclear.

Clinical studies on the cellular responses of RGCs are limited by the difficulty of acquiring RGCs from the retina. However, an alternative approach to tackle this problem is by using human induced pluripotent stem cells (hiPSCs), which represent a powerful tool for producing functional cells and tissues for functional study and in vitro disease modeling. Several efforts have been made to generate RGCs from hiPSCs for optic neuropathy studies, such as those of glaucoma [17, 18], Best disease [19], and retinitis pigmentosa [20, 21]. However, several RGC differentiation protocols yield a mixed retinal cell population containing a low proportion of RGCs [22]. To investigate the role of TRPV1 in RGC cellular response under exposure to stress, we aimed to establish an hiPSC-derived single RGC protocol to enrich the RGC population in a heterogeneous population of retinal cells.

Overall, this study aimed to (1) enrich the RGC population in hiPSC differentiation, and (2) investigate the role of TRPV1 in the osmotic stress response of RGCs.

## Methods

### Culture of hiPSCs and differentiation to RGCs

Human peripheral blood mononuclear cells (PBMCs) were collected from a healthy individual and used to generate hiPSCs. PBMCs were infected with retroviruses expressing human transcription factors OCT4, SOX2, KLF4, and c-MYC to induce pluripotency. Cell transfection was performed in DMEM/F12 medium in a culture dish pre-coated with Geltrex for 2 h. The hiPSCs were maintained in a mTeSR1 medium (STEMCELL Technologies), incubated with 5% CO_2_ at 37 °C, and passaged every 3 to 5 days.

The protocol for the differentiation of hiPSCs to RGCs was adapted with modifications from Riazifar et al. [23]. In brief, the confluent hiPSCs were scraped into small aggregates and transferred to nonadherent plates in DMEM/F12 supplemented with 1× NEAA, 1× L-glutamine, and 1× penicillin/streptomycin for 10 days to generate embryoid bodies (EBs). The medium was refreshed every 2 days. The EBs were transferred to 1% gelatin-pretreated plates and cultured in an EB medium containing 10% FBS and insulin growth factor for 7 days until neural rosettes (NRs) appeared. Subsequently, the NRs were mechanically lifted with a syringe needle and a pipette tip and grown in suspension in an EB medium containing 10% FBS and 10 mM DAPT (Merck) for another week to allow the formation of optic vesicles (OVs). The OVs were treated with trypsin-EDTA for 6 min to disperse the complex structure. The single heterogeneous cells were strained through a 40-μm strainer and plated onto a Geltrex-coated plate for 30 min to allow the attachment of the heterogeneous population. The RGC-enriched supernatant was collected, transferred to poly-d-lysine- and laminin-coated plates, and maintained in a neurobasal medium supplemented with B-27 and 10 mM DAPT for the first 5 days, after which the medium was replaced with neurobasal/B-27 and grown for other 15 days.

### Western blot

Cell samples were scraped from the culture dishes and lysed in ice-cold radioimmunoprecipitation assay (RIPA) buffer (Thermo Fisher Scientific) supplemented with 1% protease inhibitor. The cell lysate was centrifuged at 13,000 rpm for 10 min at 4 °C. The supernatant was collected for protein concentration measurement using Bradford assay. Equal weights of total proteins were separated by SDS/PAGE. The proteins were then transferred onto a polyvinylidene difluoride membrane (Millipore). The blots were incubated with blocking buffer (1× TBST and 5% skim milk) for 1 h at room temperature and then hybridized with primary antibodies overnight at 4 °C. The samples were further washed in TBST and incubated with horseradish peroxidase-conjugated secondary antibody for 1 h at room temperature. The blots were detected using X-ray film exposure.

### Immunofluorescence

Cells were washed with phosphate-buffered saline (PBS) and then fixed with 4% paraformaldehyde (Sigma-Aldrich) for 30 min at room temperature. After being washed twice with PBS, the cells were incubated with blocking buffer (10% goat serum and 0.3% Triton X-100 in PBS) for 1 h. Primary antibodies were diluted in blocking buffer and then added to the cells for 2 h at room temperature. The cells were then subjected to three 3-min washes in PBS, incubated with secondary antibodies, and diluted in blocking buffer containing DAPI (1:5000, Thermo Fisher Scientific) for 1 h at room temperature. After the 3 washes, the cells were visualized using LSM 700 confocal microscope (ZEISS).

### Intracellular and surface TRPV1 staining

For the staining of cell surface TRPV1, the paraformaldehyde-fixed RGCs were stained with rabbit anti-TRPV1 primary antibody (1:100, LifeSpan BioSciences) for 1 h and Alexa Fluor 647-conjugated anti-rabbit IgG secondary antibody (1:300, Thermo Fisher Scientific) for 1 h. The samples were then permeabilized using 0.3% Triton X-100 for 10 min after 4 washes in PBS. After additional 3 washes in PBS, the samples were further stained with rabbit anti-TRPV1 primary antibody for 1 h and Alexa Fluor 488-conjugated anti-rabbit IgG secondary antibody (1:500, Thermo Fisher Scientific) for 1 h to detect intracellular TRPV1. Nuclei were stained with DAPI (1:5000, Sigma-Aldrich), and the stained samples were examined by laser scanning microscopy using a LSM 880 microscope (ZEISS). The fluorescence intensity was quantified using ImageJ software.

### Electrophysiological analysis

The cell culture medium was replaced with artificial cerebrospinal fluid containing the following: 125 mM NaCl, 25 mM NaHCO_3_, 1.25 mM NaH_2_PO_4_, 2.5 mM KCl, 25 mM glucose, 2 mM CaCl_2_, and 1 mM MgCl_2_. Cell-attached and whole-cell recordings were performed with patch pipettes (3 to 5 MΩ) pulled from borosilicate glass tubing (outer diameter = 1.5 mm, inner diameter = 0.86 mm; Harvard Apparatus) filled with internal solution containing the following: 142 mM K-gluconate, 2 mM KCl, 0.2 mM EGTA, 4 mM Mg-ATP, 10 mM HEPES, and 7 mM Na_2_-phosphocreatine; KOH was used to adjust the pH to 7.3. Electrical activity signals of RGCs were recorded with MultiClamp 700B amplifiers or Axopatch 200B amplifiers (Molecular Devices). Data were filtered at 5 kHz and sampled at 10 kHz with a Digidata 1440A interface (Molecular Devices) controlled by pCLAMP version 10.2 (Molecular Devices). The recording temperature was 22 to 24 °C.

### ELISA

The conditioned medium derived from RGCs incubated with various concentrations of NaCl and H89 was collected and concentrated for released brain-derived neurotrophic factor (BDNF) measurements. BDNF was detected using a BDNF Human ELISA Kit (Abcam). In brief, 100 μL of the concentrated conditioned medium was added to assay plates and incubated for 2.5 h at room temperature. After washing 4 times in buffer, the antigen-conjugated ELISA plate was incubated with 100 μL of biotinylated anti-human BDNF detector for 1 h at room temperature. The samples further received 45-min incubation in HRP–streptavidin at room temperature. Finally, the products could be detected through reaction with TMB one-step substrate reagents for 30 min at room temperature in the darkness. After stop solution was administered, yellow formazan was determined using Infinite M1000 Pro plate reader (Tecan) at an absorbance of 450 nm. The released BDNF concentrations of each sample could be evaluated according to the readings of the serially diluted standard solution.

### LDH cytotoxicity assay

Lactate dehydrogenase (LDH) is known as the signal representing the integrity of cells’ plasma membranes. First, the iPSC–RGCs were maintained in 24 wells for 15 days and then added to 0/30/50/70 mM NaCl combined with or without a neurobasal medium containing 5 μM H89 for 24 h. The sample medium was collected and applied to the CytoTox 96 Non-Radioactive Cytotoxicity Assay kit (Promega) for measuring LDH activity. According to the manufacturer’s instructions, 50 μL of the sample medium and the positive control were transferred to a 96-well flat-bottom plate and gently mixed with substrate mixtures at room temperature for 30 min. Each of the reactions was terminated using 50 μL of stop solution. The LDH activity was represented by highly colored and soluble formazan with absorbance at 490 nm, which was determined by Infinite M1000 Pro plate reader (Tecan). To specifically calculate the LDH release levels in each sample, we harvested the remaining cells for protein measurement. The cells were lysed in 1× RIPA buffer and the protein concentration was measured through Braford’s method. After the protein concentration of each sample was found, the LDH release level was eventually determined as OD values/protein concentrations.

### Statistical analysis

Statistical differences between the various groups were measured using analysis of variance. A *P* value < .05 was considered significantly different. All statistical analyses were conducted using the SPSS 11.5 statistical software package (SAS, Cary, NC). All data were expressed as mean with standard deviation error bars.

## Results

### Generation of hiPSC-derived RGCs

**Fig. 1:**
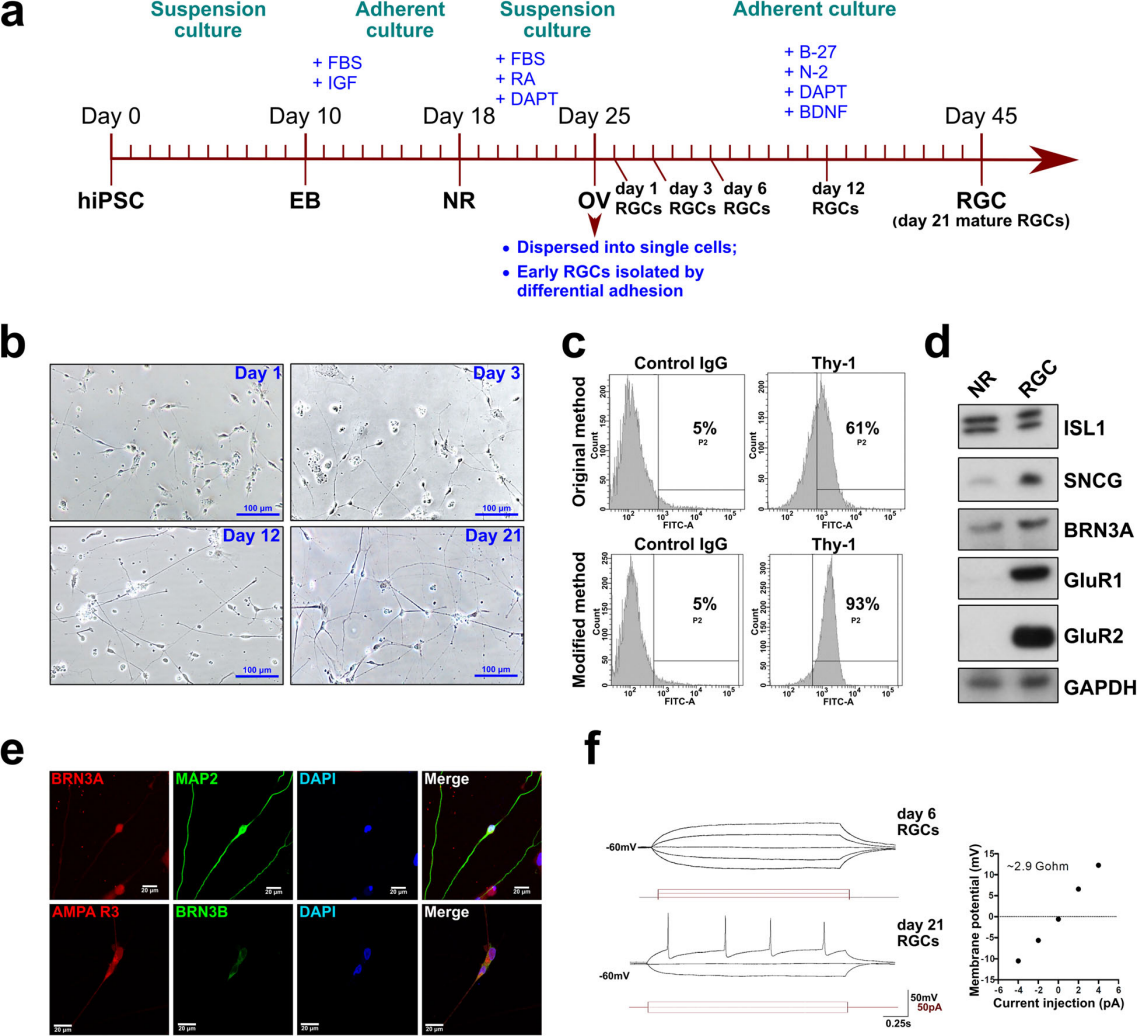
Generation of hiPSC-derived RGCs. **a** Schematic of the protocol for differentiating hiPSCs into RGCs. Culturing conditions (suspension or adherent) are shown at the top in green, crucial morphogenic factors shown at the top in blue, the intermediate stages EB—embryoid bodies, NR—neural rosettes, and OV—optic vesicles. **b** Bright-field images of RGCs after dissociation from optic vesicles from day 1 to day 21. **c** Flow cytometry analysis of the proportion of Thy-1-expressing cells in RGC populations differentiated by original and modified methods. **d** Western blot analysis demonstrating expression of positive RGC markers in mature day 21 hiPSC-derived RGCs as compared to neural rosette (NR) stage. **e** Expression of positive RGC markers in the mature day 21 RGCs demonstrated by immunofluorescent staining. **f** Electrophysiological analysis of the mature day 21 RGCs (bottom) compared to immature day 21 RGCs (top)

In our study, we used retinal ganglion cells (RGCs) derived from human-induced pluripotent stem cells (hiPSCs). To differentiate hiPSCs into RGCs, we followed a stepwise protocol of applying different culturing conditions and morphogenic factors at appropriate times, as outlined in Fig. 1a. First, embryoid bodies (EBs) were obtained by culturing hiPSCs in suspension culture, which were further differentiated into neural rosettes (NRs) and optic vesicles (OVs). Since OVs contain progenitors of different types of retinal cells, their further differentiation usually gives rise to the heterogeneous cell population. Therefore, in order to enrich the differentiation product with RGCs, we used the fact that RGCs are the first cell type that differentiates within OVs. For this reason, we purified young RGCs by dispersing OVs into separate cells with trypsin, and using the differential adhesion properties of different cell types: the trypsinized OV cells were seeded briefly onto a Geltrex-coated plate, and less adherent young RGCs were collected from the supernatant and attached other cell types were discarded (Fig. 1a). These young RGCs were further directed to differentiate into mature RGCs in the course of 3 weeks. After Geltrex selection, the purified single RGCs cultivated on the laminin-coated plate developed a harmonized typical neuronal morphology (Fig. 1a, b). RGC axon branching and lengthening were clearly observed after day 3, and mature morphology was found at approximately day 12 (Fig. 1b). As was shown by flow cytometry analysis of cells expressing the Thy-1 marker, the modified method resulted in 93% pure Thy-1-expressing RGC population as compared to 61% resultant from original unmodified method (Fig. 1c). Western blot analysis revealed the expression of the canonical RGC markers in the mature RGCs, such as Islet-1 (ISL1), γ-synuclein (SNCG), BRN3A, AMPA R1 (GluR1), and AMPA R2 (GluR2) (Fig. 1d). In addition, the expression of the RGC markers BRN3A, BRN3B, MAP2, and AMPA R3 (GluR3) was confirmed by immunofluorescent staining (Fig. 1e). The electrophysiological properties of the differentiated RGCs were further tested using the patch clamp technique. The electrophysiology profile indicated that the 6-day RGCs showed no firing pattern, which was consistent with their immature stage (Fig. 1f). On the other hand, 21-day RGCs fired the high amplitude action potential, suggesting that they are functional mature RGCs. Taken together, using our modified protocol of hiPSC differentiation, we have obtained homogeneous and functional RGC population.

### Hyperosmotic stress responses of hiPSC-derived RGCs

**Fig. 2:**
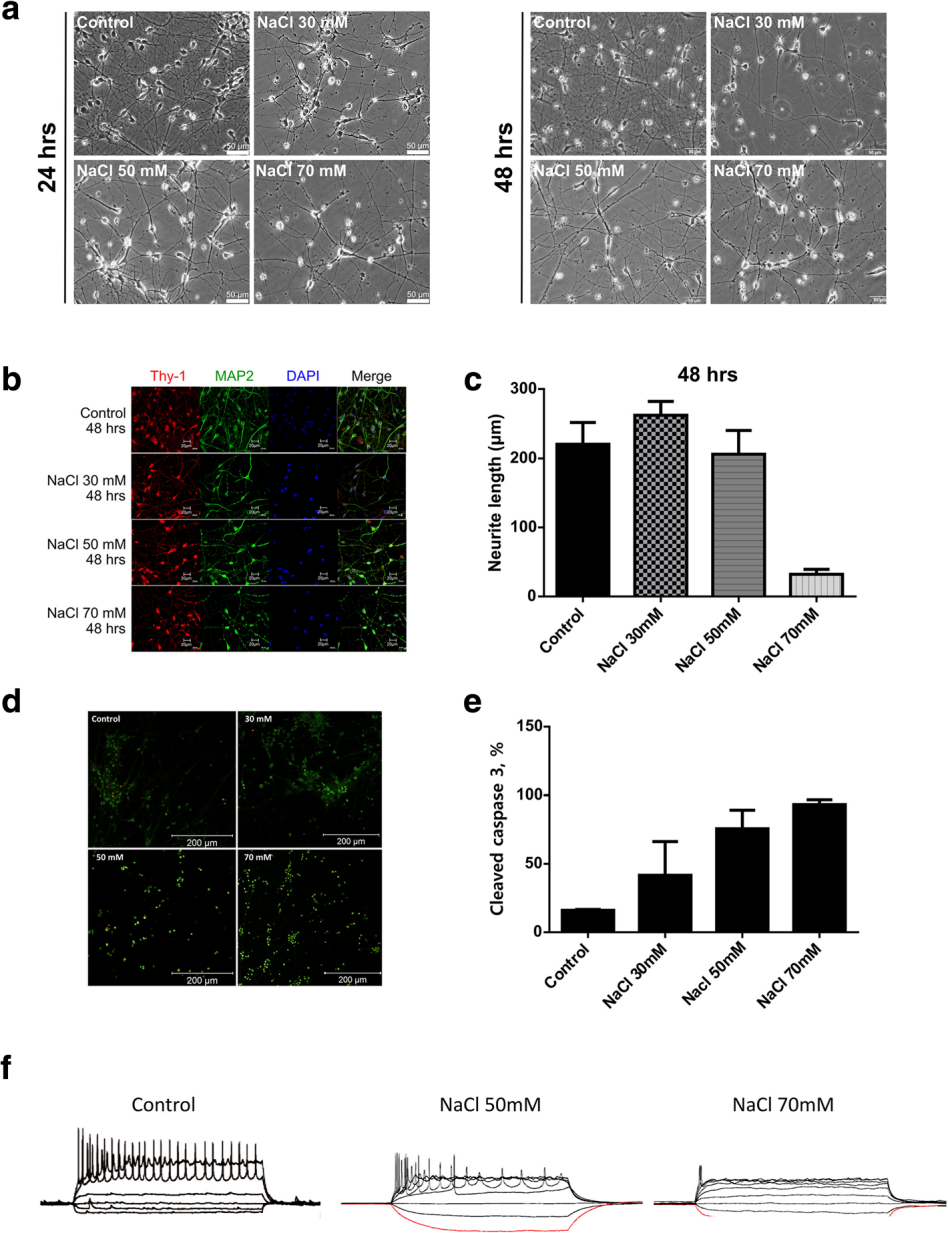
Hyperosmotic stress responses of hiPSC-derived RGCs. **a** Morphology changes of hiPSC-derived RGCs treated with various concentrations of NaCl for 24 and 48 h. **b** Immunofluorescence staining of neurite markers Thy-1 and MAP2 of hiPSC-derived RGCs treated with various concentrations of NaCl for 48 h. **c** ImageJ-measured axon lengths of iPSC-derived RGCs treated with various concentrations of NaCl for 24 h. **d** Expression of cleaved caspase 3 determined by immunostaining in hiPSC-derived RGCs incubated in the indicated concentrations of NaCl for 48 h. **e** Quantification of expression of cleaved caspase 3 in hiPSC-derived RGCs treated the indicated concentrations of NaCl for 48 h. **f** Electrophysiological analysis of RGCs treated with the indicated concentrations of NaCl for 48 h

To investigate the responses of hiPSC-derived RGCs to hyperosmotic stress, they were exposed to different concentrations of NaCl (30 mM, 50 mM, and 70 mM) for 24 and 48 h. After exposure to the hyperosmolar medium for 24 h, significant morphology changes were noted at 70 mM NaCl concentration (Fig. 2a). At the same time, significant morphology changes were apparent in hiPSC-derived RGCs treated with 30 mM, 50 mM, and 70 mM NaCl for 48 h (Fig. 2a). Immunostaining of neurites of hiPSC-derived RGCs with antibodies for Thy-1 and MAP2 revealed highly punctuate morphology after treatment with 50 mM and 70 mM NaCl for 48 h (Fig. 2b). At the same time, the neurite lengths were significantly reduced in RGCs treated with 70 mM NaCl for 48 h (Fig. 2c). To evaluate whether hyperosmotic stress accelerates cell death of RGCs, apoptosis marker, cleaved caspase 3, was detected by immunofluorescent staining. The treatment with different concentrations of NaCl for 48 h revealed proportionate increase of cleaved caspase 3 expression, which was close to 100% after treatment with 70 mM NaCl (Fig. 2d, e). At the same time, electrophysiological activity of RGCs was significantly impaired by treatment with 50 mM NaCl and completely ablated by 70 mM NaCl, as was shown by action potential measurement by patch clamp (Fig. 2f).

### TRPV1 is stimulated by NaCl-induced hyperosmotic stress in hiPSC-derived RGCs

**Fig. 3:**
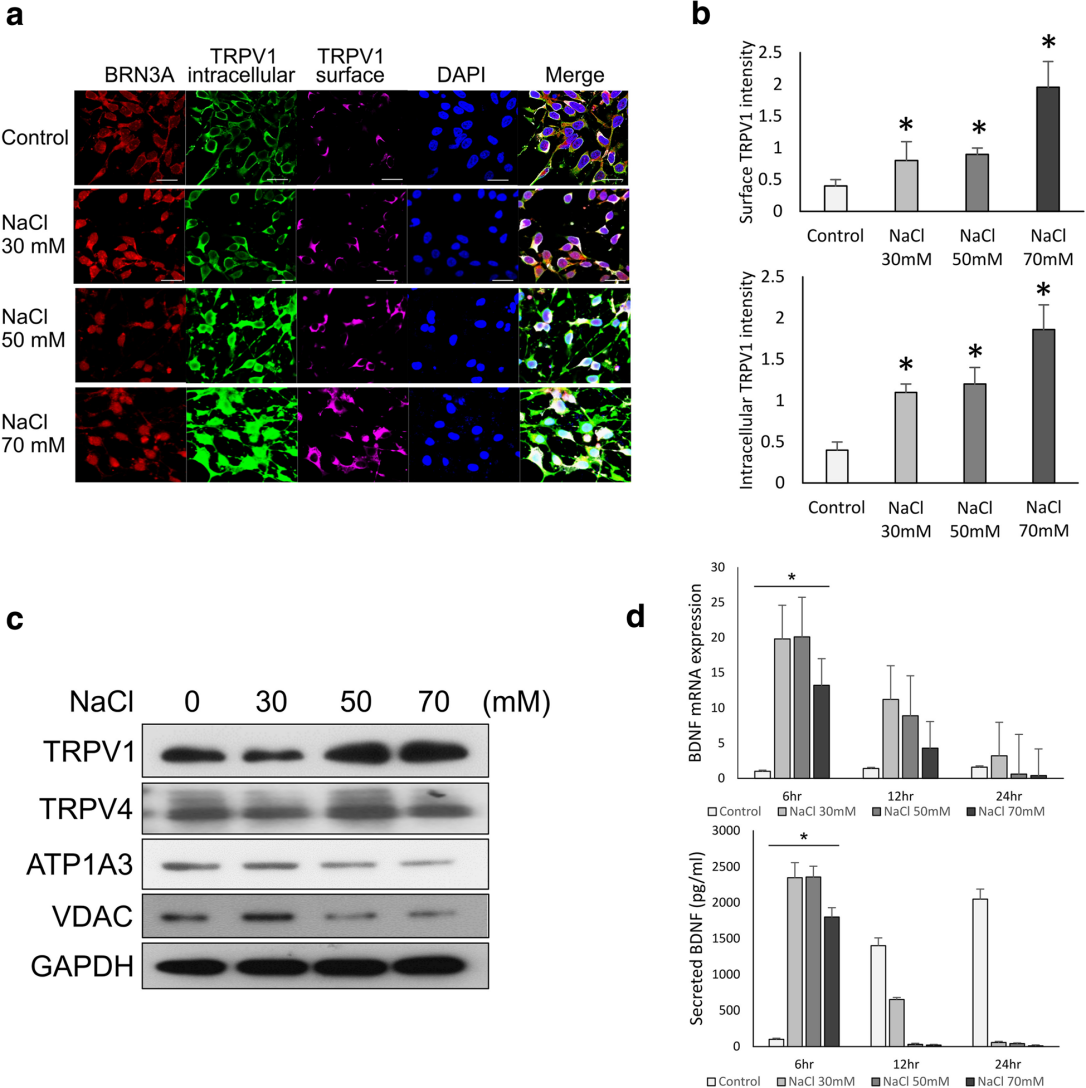
TRPV1 is stimulated by NaCl-induced hyperosmotic stress in hiPSC-derived RGCs. **a** Immunofluorescence staining of intracellular and surface TRPV1 in RGCs treated with the indicated concentrations of NaCl. DAPI used as nuclear stain. **b** ImageJ quantification of immunofluorescence intensity of intracellular and surface TRPV1 expression in RGCs treated with the indicated NaCl concentrations. Means from 3 independent measurements are shown with SD error bars. *P < 0.05, Student’s *t* test. **c** Western blot analysis of expression of TRPV1 and other ion channels in RGCs treated with the indicated concentrations of NaCl. GAPDH used as loading control. **d** qRT-PCR analysis of *BDNF* mRNA (top) and ELISA analysis of secretion of BDNF protein in RGCs treated with the indicated concentrations of NaCl for 6, 12, and 24 h. Means from 3 independent measurements are shown with SD error bars

TRPV family members have been reported to function as osmosensors that contribute to osmosensory signaling in neurons [24, 25]. TRPV1 is involved in neuronal network formation and synapse modulation [26]. It has been shown to be involved in RGC function and survival [10, 27]. To test the role of TRPV1 in the molecular response of RGCs to NaCl-induced extracellular hyperosmolarity, we analyzed TRPV1 expression at different NaCl concentrations. The TRPV1 channel was reported to localize in both plasma membrane (surface) and organellar membranes (intracellular). In this study, immunostaining suggested that both surface and intracellular TRPV1 expression levels were enhanced after treatment with NaCl (Fig. 3a). The ImageJ-quantified signal intensity of both surface and intracellular TRPV1 significantly increased, particularly after the treatment with 70 mM NaCl (Fig. 3b).

TRPV1 was reported to colocalize with TRPV4 in RGC bodies and form a protein complex in the retina [27]. Once colocalized, their expression was inversely related to high osmotic stress [27]. Through western blot analysis, we found that only TRPV1 was upregulated in the cells treated with 50 to 70 mM NaCl. No significant differential expression of TRPV4 was observed (Fig. 3c). Furthermore, other ion channels, including the Na^+^/K^+^ channels ATP1A3 and VDAC, were not dominantly altered under NaCl treatment. These results supported the crucial role of TRPV1 in RGC response under acute hyperosmotic stress induced by a NaCl solution.

Brain-derived neurotrophic factor (BDNF) is a crucial neurotrophin that supports RGC survival and function [28, 29]. Therefore, we investigated the correlation between BDNF expression and secretion, TRPV1 expression, and RGC function loss under NaCl treatment through quantitative polymerase chain reaction (qPCR) and ELISA (Fig. 3d). We found that the control RGCs increased their BDNF secretion in a time-dependent manner from 6 to 24 h, whereas RGCs treated with NaCl for 6 h exhibited a significant surge of BDNF mRNA expression and secretion in all NaCl-treated groups. High secretion of BDNF during NaCl-induced stress within a short time indicated that BDNF secretion was a protective response from the RGCs against external stress. Either mRNA expression or BDNF secretion in the treated RGCs was drastically reduced 12 to 24 h after treatment. These results implied that RGCs may secrete BDNF during osmotic stress to protect and support RGC survival; the protective response of BDNF decreased after 12 h of stress exposure.

### Effects of PKA inhibitor (H89) on NaCl-induced cell stress in hiPSC**-**derived RGCs

**Fig. 4:**
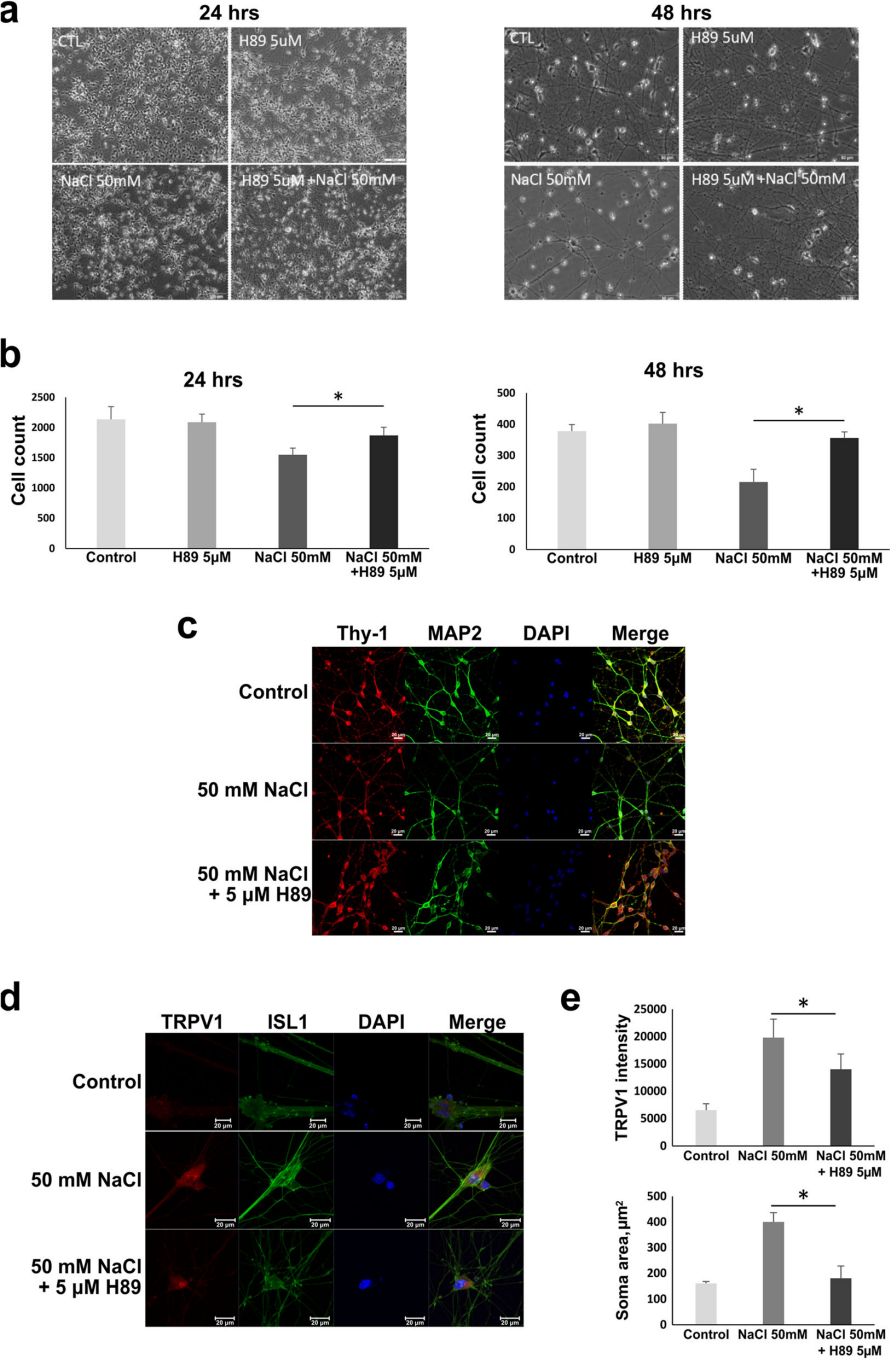
Effects of PKA inhibitor (H89) on NaCl-induced cell stress in hiPSC-derived RGCs. **a** Morphology of RGCs treated with 50 mM NaCl with or without 5 μm H89 for 24 and 48 h. **b** The numbers of cells per low-power field in **a** were counted and shown in the bar graphs. Means from 3 measurements are shown with SD error bars. *P < 0.05, Student’s *t* test. **c** Immunofluorescence staining of axonal markers Thy-1 and MAP2 in RGCs treated for 24 h with NaCl with or without H89. **d** Immunofluorescence images of RGCs treated for 24 h with NaCl with or without H89 after double staining with TRPV1 and Islet1 (ISL1) antibodies. Cells exposed to NaCl appeared with an enlarged soma, which was ameliorated in the H89-treated group. **e** The measurement of neuron soma size (area) and TRPV1 expression. Means from 3 measurements are shown with SD error bars. *P < 0.05, Student’s *t* test

TRPV1 activation is regulated by the phosphorylation and dephosphorylation processes. It was found that cAMP-dependent protein kinase A (PKA) directly phosphorylates the N-terminus of TRPV1 and controls channel sensitization [30]. Therefore, we sought to investigate the effect of PKA inhibitor H89 on TRPV1 regulation in the conditions of NaCl-induced hyperosmotic stress. We exposed RGCs to 50 mM NaCl for 24 and 48 h with or without 24 h of pretreatment with the PKA inhibitor H89. We found defective axon branching and significantly decreased cell number in NaCl-treated RGCs, in particular after 48 h of incubation (Fig. 4a, b). On the other hand, the RGCs pretreated with H89 showed less abnormality and were comparable in their morphology to the control RGCs (Fig. 4a). This result indicated that 5 μM H89 maintained cell survival and protected RGCs from NaCl-induced cell loss and axonopathy. We further observed RGC functional protein expression by immunostaining Thy-1 and MAP2 (Fig. 4c); their expression was noticeably reduced in RGCs incubated for 24 h in NaCl. However, the stressed RGCs pretreated with H89 showed an expression level similar to that in the cells under normal conditions. To confirm the correlations between NaCl-induced hyperosmolarity, TRPV1 expression, and the PKA inhibitor, TRPV1 expression was observed by immunostaining (Fig. 4d); TRPV1 was noticeably upregulated under NaCl treatment. Although the TRPV1 expression in H89-pretreated cells was observable, the signal intensity was significantly lower than in NaCl-treated cells (Fig. 4d, lower). This result indicated the inhibitory effect of H89 on TRPV1 activation. Notably, we observed that the soma size of NaCl-stressed RGCs was much larger than that of the control cells (Fig. 4d, e, upper), indicating cell swelling under the stress condition. However, RGCs pretreated with H89 had markedly smaller soma sizes than those without H89 pretreatment. Overall, suppression of TRPV1 activation by the PKA inhibitor was revealed to be essential for supporting RGCs to sustain the NaCl-stressed environment.

### H89 attenuates NaCl-induced hyperosmotic stress effects on hiPSC-derived RGCs

**Fig. 5:**
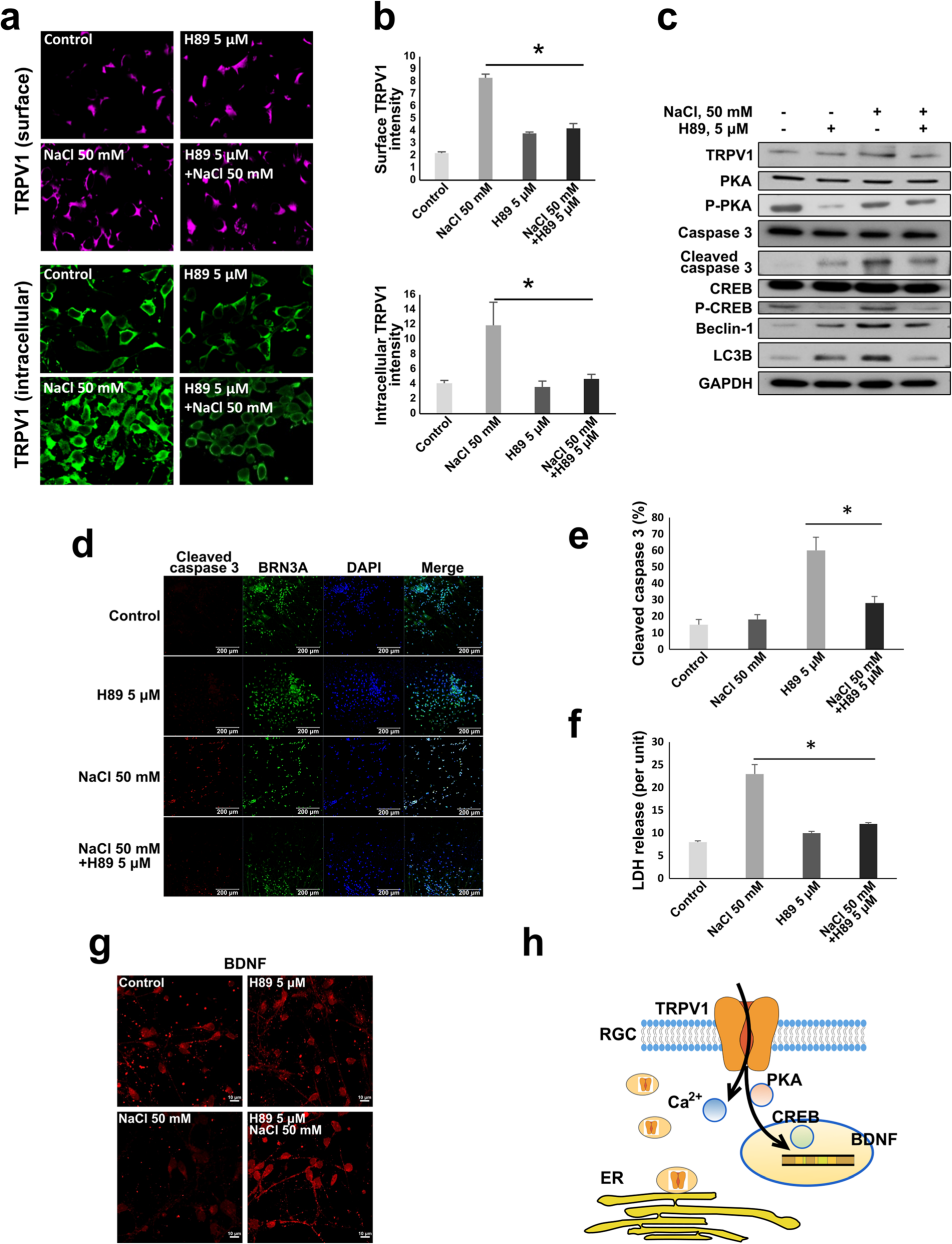
H89 attenuates NaCl-induced hyperosmotic stress effects on hiPSC-derived RGCs. **a** Immunofluorescence staining of surface and intracellular TRPV1 in hiPSC-derived RGCs treated with 50 mM NaCl with or without 5 μM H89 for 24 h. **b** The signal intensity in **a** was quantified using ImageJ and is shown in the bar graph with standard deviation. **c** Western blot profile of cellular responses in hiPSC-derived RGCs treated with 50 mM NaCl solution with or without 5 μM H89 pretreatment. **d** Immunofluorescence staining of cleaved caspase 3 performed to evaluate cell apoptosis. **e** The percentage of cleaved caspase 3-positive cells was quantified and is demonstrated in the bar graph. **f** LDH released from damaged cell was measured to evaluate the cytotoxicity; the results are demonstrated in the bar graph. **g** Immunofluorescent staining of BDNF expressed after the indicated treatments. **h** Schematic illustrating the interplay between TRPV1, PKA, CREB, and restored BDNF secretion under hyperosmotic-stress-induced damage. Asterisk (*) indicates a significant difference (*P* < .05)

The aforementioned results revealed that intracellular and cell surface localization of TRPV1 were affected by NaCl-induced hyperosmolarity. As indicated, 5 μM H89 attenuated hyperosmotic stress induced by 50 mM NaCl and maintained an intact neuron structure. Therefore, we sought to elaborate whether H89 affects the localization of TRPV1. Through immunofluorescence staining of intracellular and surface TRPV1, we showed that NaCl-induced osmotic stress enhanced TRPV1 expression in both surface and intracellular localizations (Fig. 5a). RGCs pretreated with 5 μM H89 exhibited lower TRPV1 staining signals, particularly intracellular TRPV1, when compared with cells without pretreatment (Fig. 5a). The signal intensity was quantified and is depicted in Fig. 5b. This result suggested that PKA may regulate intracellular TRPV1 activity or be related to the translocation of TRPV1 between the intracellular and cellular membranes. We subsequently explored the intracellular response pathway by evaluating the expression of the related proteins in the PKA/TRPV1 axis. Through western blot analysis (Fig. 5c), we noted that a phosphorylated form of CREB (P-CREB), which was previously reported to be a PKA target, was upregulated under both normal conditions and in NaCl-treated environment. Notably, the expression profiles of RGC molecular response to H89 treatment differed between the normal and NaCl-treated conditions. Under the normal condition, H89 had no significant effect on TRPV1 expression, but enhanced apoptosis (cleaved caspase 3) and autophagy (Beclin-1 and LC3B) protein expression (Fig. 5c). In contrast, under the NaCl-stressed condition, H89 treatment attenuated apoptosis and autophagy (Fig. 5c). Consistently, we also observed the same effect on apoptosis by immunostaining the cleaved caspase 3, i.e., we found that NaCl induced apoptosis, which was attenuated by H89 (Fig. 5d, e). Cell cytotoxicity was further determined by lactate dehydrogenase (LDH) release assay; significantly higher LDH release by RGCs under NaCl stress was attenuated by H89 pretreatment (Fig. 5f). BDNF expression, found to be reduced under the NaCl-stressed condition, was confirmed through immunostaining (Fig. 5g). Moreover, 50 mM NaCl greatly abated BDNF expression, whereas H89 increased its expression under high extracellular NaCl. Taken together, the results indicate that PKA may regulate TRPV1 intracellular localization, translocation, and CREB phosphorylation. Inhibiting PKA using H89 ameliorated NaCl-induced RGC damage by regulating BDNF expression (Fig. 5h).

## Discussion

RGCs are a vulnerable cell type. Because RGC apoptosis plays a crucial role in numerous diseases such as optic neuritis, traumatic optic neuropathy, light-induced retinopathy, ischemic RGC death, diabetic retinopathy, and glaucoma [31], recent investigations have focused on conceivable medical interventions to prevent or treat RGC loss. Some promising results have been found for numerous neuroprotective strategies or drugs in animal studies, which target various putative mechanisms. However, to date, outcomes of translational clinical trials are less encouraging. This study investigated the mechanism regulating RGC homeostasis under injury to provide a basic understanding for future therapeutic research.

TRP channels have been found to be involved in the visual signaling of retinal cells through maintaining Ca^2+^ homeostasis by means of controlling extracellular Ca^2+^ influx and intracellular Ca^2+^ stores in response of RGCs to light [32, 33]. Apart from RGCs, TRPV1 channel is present in other retina cell types such as photoreceptors, bipolar cells, amacrine cells, and glial cells. OVs, which constitute an intermediate stage of differentiation of hiPSCs to RGCs, are composed of precursors of multiple retinal cell types. Therefore, without the proper separation of these cell types, the study of RGC-specific roles of TRPV1 could be problematic due to admixture of other cell types. Although we previously successfully differentiated RGCs from OVs, a homogenous RGC population was not completely acquired. To enrich RGCs in the heterogeneous population of differentiated OVs, we applied two major modifications. Several growth factors were used to stimulate the differentiation of OVs into a neuronal lineage and maintain RGC survival. Additionally, because RGCs are the first cell type to appear in the early stage of retinal development, the RGC isolation procedure was shifted to an earlier stage and a short passage was performed on a plate coated with Geltrex to selectively screen unattached RGCs and discard attached heterogeneous cells (Fig. 1a). We showed that such modified method resulted in more pure homogeneous population as could be judged from the higher enrichment of cells expressing Thy-1 marker (Fig. 1c). After a series of validations, we found that the RGC-specific protein expression and electrophysiological activity were similar to those of RGCs generated using previously described protocols [22, 34, 35].

Hypertonic agents such as NaCl, sucrose, and urea have been used to induce hyperosmotic conditions to study various stress adaptation pathways. NaCl was previously employed to generate a hyperosmotic condition in RPE cells to observe the effect of osmotic stress on the gene expression profile of ARPE-19 [36]. In this study, we conducted NaCl administration to explore the role of TRPV1 in the morphological and physiological responses of RGCs exposed to hyperosmotic stress. We demonstrated that activation of RGC-expressed TRPV1 contributed to the cellular response in hyperosmotic stress of hiPSC-derived RGCs. A high concentration of NaCl caused hyperosmolarity, which led to defective RGC axon growth and enhancement of apoptosis and autophagy. Expression of TRPV1 was significantly upregulated under NaCl-induced hyperosmotic stress. These results suggested that RGC responded to NaCl-induced hyperosmolarity by enhancing the expression of TRPV1, and the hyperosmolarity-mediated cell death and autophagy may be partially regulated by TRPV1. A report showed that TRPV1 agonism increased RGC apoptosis at an ambient pressure and TRPV1 antagonism reduced apoptosis under elevated hydrostatic pressure [10]. Furthermore, a study on retinal explants consistently indicated that Trpv1−/− and pharmacological TRPV1 antagonism decreased pressure-induced RGC apoptosis [27]. By contrast, another in vivo study showed that pharmacological antagonism of TRPV1 accelerated neuropathy of ganglion cells in rats suffering elevated intraocular pressure [37]. Our findings support the hypothesis that TRPV1 upregulation under hyperosmotic stress increases cell apoptosis and RGC loss. Thus, inhibiting the overexpression of TRPV1 may help to protect RGCs from stress damage.

Several studies have shown that TRPV1 is phosphorylated by cAMP-dependent protein kinase A (PKA) and protein kinase C (PKC). PKA-mediated phosphorylation sensitizes the TRPV1 channel and reduces desensitization of capsaicin-activated currents [38, 39]. Similar to other members of the TRP family, activated TRPV1 induces a potent influx of extracellular Ca^2+^ into neural cells and transduces the Ca^2^-mediated signal, including Ca^2+^/calmodulin-dependent protein kinase (CaMK) [40]. CaMK, a critical protein in neuron function, activates transcription factor CREB through phosphorylation [41]. We found that TRPV1 overexpression was related to PKA activity. PKA inhibitor H89 abolished osmotic stress-induced TRPV1 upregulation (immunostaining) and suppressed phosphorylation of CREB (determined by Western blot analysis). In addition, RGC apoptosis and autophagy in response to pressure stress were rescued. These results emphasized that TRPV1 plays a crucial role in the pressure stress response of RGCs through the PKA/TRPV1/CREB axis, and inhibition of PKA abrogated TRPV1-induced RGC damage.

Inactivated PKA tetramer comprises 2 regulatory and 2 catalytic subunits. When the catalytic subunits bind ATP, they become catalytically active, which causes phosphorylation of serine and threonine residues at the target sites. H89 is an isoquinoline derivative developed from the nonspecific PKA and protein kinase G (PKG) inhibitor H8. H89 blocks PKA action through competitive inhibition of the ATP site on the PKA catalytic subunit [42]. TRPV1 and CREB are thought to be the phosphorylation targets of PKA. Therefore, inhibiting PKA could diminish phosphorylation of these two proteins. Using immunostaining and western blot analysis, we clearly demonstrated that H89 administration not only regulated TRPV1 expression, but also decreased phosphorylation of CREB. Furthermore, evidence exists that activated p-CREB modulates the expression of several target genes related to neuron function, including BDNF [43]. BDNF is a neuronal growth factor that critically stimulates neurogenesis and neuronal maturation by activating CREB and PKA [44]. In this study, ELISA results indicated that the secretion of BDNF in the control group (ambient pressure) increased in a time-dependent manner. In addition, under hyperosmotic stress, BDNF secretion dramatically surged and declined, which may be related to cell compensatory responses. Notably, BDNF was increased by the PKA inhibitor, which suppressed p-CREB. Therefore, we assume that other mediators related to the control of BDNF expression exist.

Although the TRPV family was initially recognized as a plasma membrane channel, recent studies have uncovered that localization of TRPV1 is not restricted to the cell surface; the intracellular vesicular membrane localization of TRPV1 has been discovered [19, 45]. The first evidence of intracellular TRPV1 demonstrated the localization of heterologously expressed rat TRPV1 on the endoplasmic reticulum (ER) in monkey fibroblast-like cell line COS-7 [46]. TRPV1 has been identified in several organelles, such as the ER [47], sarcoplasmic reticulum [48], mitochondria [48], Golgi complex, and lysosomes [49] of several cell types, including HEK 293, HeLa, DRG, skeletal myocyte, and microglia, as well as several cancer cells. Organellar Ca^2+^ homeostasis is essential for cell function. Aberrant Ca^2+^ regulation was documented to trigger ER stress, which leads to apoptosis [50]. Similarly, disrupting Ca^2+^ homeostasis in mitochondria causes several pathological conditions [51]. In our study, we noted that the intracellular localization of TRPV1 was increased in a high NaCl environment. In addition, cell apoptosis and autophagy were enhanced. Given that the TRPV1 channel corresponds to the regulation of Na^+^ and Ca^2+^ influx and may serve as an ER Ca^2+^ release channel whose activation leads to ER stress, our study may support these data. However, we did not specifically identify the organellar localization of TRPV1. Coimmunostaining of TRPV1 and organelle markers or cell fractionation may be useful for more in-depth understanding of intracellular TRPV1 behavior in osmotically stressed RGCs [52].

## Conclusions

In conclusion, we successfully modified the RGC differentiation protocol to enrich the RGC population from a heterogeneous cell population of OVs. A high concentration of NaCl constituted the hyperosmolarity milieu for iPSC–RGCs and activated the TRPV1 channel. Overexpression of TRPV1 enhanced ion influx, which was followed by activation of apoptosis, autophagy cascade, and functional loss of RGCs. Furthermore, PKA regulated TRPV1 activation, localization, and translocation between the intracellular and cellular membranes. PKA inhibitor H89 supported the balancing of TRPV1 expression and translocation under NaCl-induced osmotic stress. BDNF, a front-line neurotrophin released to support RGC survival, was simultaneously maintained by H89. Our findings of TRPV1’s role and PKA-mediated RGC rescue under hyperosmolarity provide a new option for the future development of therapies in eye diseases involving RGC loss.

## Data Availability

The datasets used and/or analyzed during the current study are available from the corresponding author on reasonable request.

## Abbreviations

BDNF: Brain-derived neurotrophic factor
CREB: cAMP response element binding
hiPSC: Human-induced pluripotent stem cell
LDH: Lactate dehydrogenase
NR: Neural rosette
OV: Optic vesicle
PKA: Protein kinase A
RGC: Retinal ganglion cells
TRPV1: Transient receptor potential vanilloid 1
